# Supplementary material for: Modeling glioblastoma heterogeneity as a dynamic network of cell states
Source: Mol Syst Biol. 2021 Sep 16;17(9):e10105. doi: 10.15252/msb.202010105 (PMC8444284; doi:10.15252/msb.202010105)
Supplement: Supplementary file 5 — Source Data for Figure 3 [file MSB-17-e10105-s001.zip › Figure3A_sourcedata/GSEA_3065/nosigdb_state2.GseaPreranked.1622718419215/SEURAT_CELLCYCLE.html]

Details for gene set SEURAT\_CELLCYCLE[GSEA]

|  || Dataset | state2 |
| Phenotype | NoPhenotypeAvailable |
| Upregulated in class | na\_pos |
| GeneSet | SEURAT\_CELLCYCLE |
| Enrichment Score (ES) | 0.8984652 |
| Normalized Enrichment Score (NES) | 2.9555845 |
| Nominal p-value | 0.0 |
| FDR q-value | 0.0 |
| FWER p-Value | 0.0 |
Table: GSEA Results Summary

  

Fig 1: Enrichment plot: SEURAT\_CELLCYCLE      
 Profile of the Running ES Score & Positions of GeneSet Members on the Rank Ordered List

  

| PROBE | GENE SYMBOL | GENE\_TITLE | RANK IN GENE LIST | RANK METRIC SCORE | RUNNING ES | CORE ENRICHMENT || 1 | HMGB2 |  |  | 4 | 0.823 | 0.0272 | Yes |
| 2 | TYMS |  |  | 5 | 0.792 | 0.0537 | Yes |
| 3 | UBE2C |  |  | 9 | 0.704 | 0.0770 | Yes |
| 4 | TOP2A |  |  | 15 | 0.596 | 0.0965 | Yes |
| 5 | PCNA |  |  | 16 | 0.595 | 0.1164 | Yes |
| 6 | MKI67 |  |  | 17 | 0.590 | 0.1362 | Yes |
| 7 | CDK1 |  |  | 18 | 0.577 | 0.1556 | Yes |
| 8 | ATAD2 |  |  | 21 | 0.563 | 0.1743 | Yes |
| 9 | RRM2 |  |  | 22 | 0.556 | 0.1929 | Yes |
| 10 | CKS1B |  |  | 23 | 0.554 | 0.2115 | Yes |
| 11 | CLSPN |  |  | 27 | 0.536 | 0.2291 | Yes |
| 12 | CKS2 |  |  | 28 | 0.532 | 0.2470 | Yes |
| 13 | TPX2 |  |  | 29 | 0.531 | 0.2648 | Yes |
| 14 | CENPF |  |  | 34 | 0.518 | 0.2818 | Yes |
| 15 | GINS2 |  |  | 37 | 0.506 | 0.2985 | Yes |
| 16 | SMC4 |  |  | 42 | 0.489 | 0.3145 | Yes |
| 17 | TMPO |  |  | 43 | 0.489 | 0.3309 | Yes |
| 18 | NUSAP1 |  |  | 47 | 0.483 | 0.3468 | Yes |
| 19 | RFC2 |  |  | 53 | 0.460 | 0.3617 | Yes |
| 20 | UHRF1 |  |  | 57 | 0.454 | 0.3766 | Yes |
| 21 | BIRC5 |  |  | 61 | 0.443 | 0.3911 | Yes |
| 22 | GMNN |  |  | 64 | 0.435 | 0.4055 | Yes |
| 23 | RRM1 |  |  | 72 | 0.412 | 0.4185 | Yes |
| 24 | MCM4 |  |  | 79 | 0.399 | 0.4313 | Yes |
| 25 | MCM5 |  |  | 83 | 0.394 | 0.4442 | Yes |
| 26 | GTSE1 |  |  | 89 | 0.389 | 0.4567 | Yes |
| 27 | RAD51AP1 |  |  | 90 | 0.388 | 0.4697 | Yes |
| 28 | CDC45 |  |  | 95 | 0.377 | 0.4819 | Yes |
| 29 | CENPE |  |  | 105 | 0.372 | 0.4934 | Yes |
| 30 | CDC6 |  |  | 106 | 0.372 | 0.5059 | Yes |
| 31 | FEN1 |  |  | 107 | 0.371 | 0.5183 | Yes |
| 32 | ANLN |  |  | 111 | 0.358 | 0.5300 | Yes |
| 33 | SLBP |  |  | 112 | 0.357 | 0.5420 | Yes |
| 34 | NDC80 |  |  | 115 | 0.354 | 0.5537 | Yes |
| 35 | KIF23 |  |  | 117 | 0.351 | 0.5653 | Yes |
| 36 | POLD3 |  |  | 120 | 0.347 | 0.5767 | Yes |
| 37 | TUBB4B |  |  | 125 | 0.340 | 0.5877 | Yes |
| 38 | KIF11 |  |  | 129 | 0.335 | 0.5986 | Yes |
| 39 | TACC3 |  |  | 130 | 0.334 | 0.6098 | Yes |
| 40 | ANP32E |  |  | 136 | 0.330 | 0.6204 | Yes |
| 41 | USP1 |  |  | 138 | 0.329 | 0.6313 | Yes |
| 42 | DTL |  |  | 148 | 0.311 | 0.6408 | Yes |
| 43 | MCM6 |  |  | 149 | 0.311 | 0.6512 | Yes |
| 44 | CDCA2 |  |  | 150 | 0.309 | 0.6616 | Yes |
| 45 | CKAP2 |  |  | 151 | 0.308 | 0.6719 | Yes |
| 46 | NASP |  |  | 156 | 0.306 | 0.6818 | Yes |
| 47 | CDCA3 |  |  | 160 | 0.304 | 0.6917 | Yes |
| 48 | HELLS |  |  | 161 | 0.304 | 0.7019 | Yes |
| 49 | AURKB |  |  | 163 | 0.303 | 0.7119 | Yes |
| 50 | ECT2 |  |  | 171 | 0.297 | 0.7211 | Yes |
| 51 | NUF2 |  |  | 188 | 0.287 | 0.7290 | Yes |
| 52 | CCNB2 |  |  | 190 | 0.286 | 0.7385 | Yes |
| 53 | CDC20 |  |  | 193 | 0.284 | 0.7479 | Yes |
| 54 | KIF20B |  |  | 208 | 0.278 | 0.7557 | Yes |
| 55 | HJURP |  |  | 209 | 0.277 | 0.7650 | Yes |
| 56 | CBX5 |  |  | 225 | 0.265 | 0.7723 | Yes |
| 57 | MCM2 |  |  | 228 | 0.262 | 0.7809 | Yes |
| 58 | CENPA |  |  | 238 | 0.259 | 0.7886 | Yes |
| 59 | KIF2C |  |  | 252 | 0.251 | 0.7956 | Yes |
| 60 | MSH2 |  |  | 266 | 0.244 | 0.8024 | Yes |
| 61 | CCNE2 |  |  | 288 | 0.236 | 0.8081 | Yes |
| 62 | CKAP2L |  |  | 327 | 0.224 | 0.8116 | Yes |
| 63 | BUB1 |  |  | 335 | 0.222 | 0.8183 | Yes |
| 64 | BLM |  |  | 355 | 0.215 | 0.8234 | Yes |
| 65 | HMMR |  |  | 358 | 0.215 | 0.8304 | Yes |
| 66 | RPA2 |  |  | 367 | 0.212 | 0.8367 | Yes |
| 67 | DLGAP5 |  |  | 371 | 0.211 | 0.8434 | Yes |
| 68 | AURKA |  |  | 375 | 0.210 | 0.8502 | Yes |
| 69 | DSCC1 |  |  | 395 | 0.202 | 0.8549 | Yes |
| 70 | UNG |  |  | 398 | 0.200 | 0.8614 | Yes |
| 71 | BRIP1 |  |  | 426 | 0.194 | 0.8650 | Yes |
| 72 | NCAPD2 |  |  | 434 | 0.193 | 0.8707 | Yes |
| 73 | TIPIN |  |  | 440 | 0.192 | 0.8766 | Yes |
| 74 | UBR7 |  |  | 458 | 0.189 | 0.8811 | Yes |
| 75 | CKAP5 |  |  | 596 | 0.164 | 0.8720 | Yes |
| 76 | CASP8AP2 |  |  | 608 | 0.162 | 0.8763 | Yes |
| 77 | CDCA8 |  |  | 634 | 0.158 | 0.8789 | Yes |
| 78 | E2F8 |  |  | 638 | 0.158 | 0.8838 | Yes |
| 79 | EXO1 |  |  | 643 | 0.157 | 0.8887 | Yes |
| 80 | PRIM1 |  |  | 660 | 0.155 | 0.8922 | Yes |
| 81 | CTCF |  |  | 691 | 0.151 | 0.8940 | Yes |
| 82 | WDR76 |  |  | 702 | 0.150 | 0.8980 | Yes |
| 83 | POLA1 |  |  | 752 | 0.143 | 0.8976 | Yes |
| 84 | NEK2 |  |  | 805 | 0.138 | 0.8966 | Yes |
| 85 | TTK |  |  | 831 | 0.135 | 0.8985 | Yes |
| 86 | PSRC1 |  |  | 1023 | 0.118 | 0.8820 | No |
| 87 | RANGAP1 |  |  | 1157 | 0.107 | 0.8714 | No |
| 88 | GAS2L3 |  |  | 1326 | 0.096 | 0.8566 | No |
| 89 | CHAF1B |  |  | 1530 | 0.085 | 0.8377 | No |
| 90 | CDCA7 |  |  | 1684 | 0.078 | 0.8240 | No |
| 91 | CDC25C |  |  | 1820 | 0.072 | 0.8120 | No |
| 92 | G2E3 |  |  | 2405 | 0.051 | 0.7512 | No |
| 93 | LBR |  |  | 7064 | -0.038 | 0.2542 | No |
Table: GSEA details [plain text format]

  

Fig 2: SEURAT\_CELLCYCLE: Random ES distribution      
 Gene set null distribution of ES for **SEURAT\_CELLCYCLE**
